# Enhancing energy and glucose metabolism by disrupting triglyceride synthesis: Lessons from mice lacking DGAT1

**DOI:** 10.1186/1743-7075-3-10

**Published:** 2006-01-31

**Authors:** Hubert C Chen

**Affiliations:** 1 Department of Medicine, University of California, San Francisco, CA 94103, USA; 2 Department of Medical Sciences, Amgen Inc., Thousand Oaks, CA 91320, USA

## Abstract

Although the ability to make triglycerides is essential for normal physiology, excess accumulation of triglycerides results in obesity and is associated with insulin resistance. Inhibition of triglyceride synthesis, therefore, may represent a feasible strategy for the treatment of obesity and type 2 diabetes. Acyl CoA:diacylglycerol acyltransferase 1 (DGAT1) is one of two DGAT enzymes that catalyze the final reaction in the known pathways of mammalian triglyceride synthesis. Mice lacking DGAT1 have increased energy expenditure and insulin sensitivity and are protected against diet-induced obesity and glucose intolerance. These metabolic effects of DGAT1 deficiency result in part from the altered secretion of adipocyte-derived factors. Studies of DGAT1-deficient mice have helped to provide insights into the mechanisms by which cellular lipid metabolism modulates systemic carbohydrate and insulin metabolism, and a better understanding of how DGAT1 deficiency enhances energy expenditure and insulin sensitivity may identify additional targets or strategies for the treatment of obesity and type 2 diabetes.

## Introduction

Triglycerides (triacylglycerols) are neutral lipids consisting of a glycerol backbone and three long-chain fatty acids. In addition to representing a major form of stored energy in the adipose tissue and skeletal muscle, triglycerides are an integral component of lipoprotein particles synthesized by the liver and small intestine, skin sebum secreted by sebaceous glands, and milk produced by mammary glands. Clearly, the ability to make triglycerides is essential for normal physiology.

Excess triglyceride accumulation, however, leads to obesity and, particularly when it occurs in non-adipose tissues, is associated with insulin resistance. Inhibition of triglyceride synthesis, therefore, represents a potential therapeutic strategy for human obesity and type 2 diabetes. Until recently, it had been unclear whether disrupting triglyceride synthesis would be beneficial or would instead cause significant adverse consequences. The generation of mice lacking acyl CoA:diacylglycerol acyltransferase 1 (DGAT1), a key enzyme in the triglyceride synthesis pathway, has helped to provide insights into the metabolic effects of inhibiting triglyceride synthesis in animals [1].

### Role of DGAT1 in triglyceride synthesis

DGAT1 is one of two DGAT enzymes that catalyze the linkage of a *sn*-1,2-diacylglycerol with a fatty acyl CoA to form a triglyceride molecule. In cells, the reaction catalyzed by DGAT1 takes place primarily in the endoplasmic reticulum. Although DGAT1 is widely expressed in tissues, the highest expression levels in humans are in tissues typically associated with triglyceride metabolism, in particular the adipose tissue, small intestine, and liver. The tissue distribution pattern is similar in humans and mice [2].

Although DGAT1 is primarily identified as a triglyceride synthesis enzyme, recent findings indicate that it is also capable of catalyzing the formation of diacylglycerols, waxes, and retinyl esters *in vitro *[3]. It will be of interest to determine the relative importance of each of these functions *in vivo*.

### Decreased adiposity and tissue triglyceride levels in DGAT1-deficient mice

DGAT1-deficient (*Dgat1*^-/-^) mice are viable and have ~50% less adipose mass and smaller adipocytes than wild-type (WT) mice on a chow diet. Triglyceride levels in the adipose tissue and skeletal muscle of *Dgat1*^-/- ^mice are also decreased by ~30–40%. Although differences in liver triglyceride levels are not statistically significant in chow-fed conditions, on a high-fat diet liver triglyceride levels are significantly lower in *Dgat1*^-/- ^mice than in WT mice [1,4].

Tissue levels of diacylglycerol, a substrate for the DGAT reaction, are not elevated, and in fact tend to be lower in the skeletal muscle and liver of *Dgat1*^-/- ^mice. These findings may be explained in part by the loss of acyl CoA:monoacylglycerol acyltransferase (MGAT) activity mediated by DGAT1 [3]. DGAT1 deficiency also alters the fatty acid composition of triglycerides in the adipose tissue and skeletal muscle, resulting in a relative decrease in monounsaturated (16:1 and 18:1) fatty acids and a relative increase in saturated (16:0 and 18:0) fatty acids [4]. Although mechanisms of this altered fatty acid composition remain incompletely understood, it may reflect a decreased utilization of saturated lipids in DGAT1-mediated triglyceride synthesis. Such substrate preference has been reported for murine MGAT2, which catalyzes the formation of diacylglycerols in the small intestine [5].

Despite the absence of DGAT1, serum triglyceride levels are normal in *Dgat1*^-/- ^mice, suggesting that either DGAT1 does not play a rate-limiting role in hepatic triglyceride secretion, or alternative mechanisms can compensate for the loss of DGAT1 to maintain normal serum triglyceride levels [1]. Serum levels of free fatty acids are also similar in WT and *Dgat1*^-/- ^mice.

### Increased insulin sensitivity in DGAT1-deficient mice

**Figure 1:**
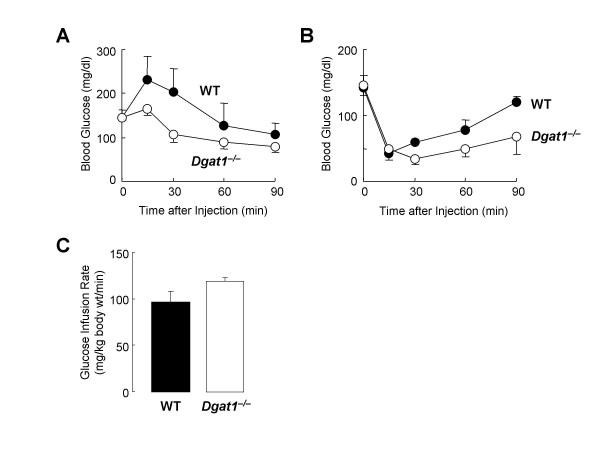
**Increased insulin sensitivity in *Dgat1*^-/- ^mice**. A. Glucose tolerance test. B. Insulin tolerance test. C. Hyperinsulinemic-euglycemic clamp study. Reproduced with permission from [3].

Correlating with the decreased adiposity, glucose metabolism is enhanced in *Dgat1*^-/- ^mice, as evidenced by decreased blood glucose concentrations after either an intraperitoneal glucose load (Figure 1A) or an insulin injection (Figure 1B) in *Dgat1*^-/- ^mice. An increase in insulin sensitivity is confirmed by results from hyperinsulinemic-euglycemic clamp studies (Figure 1C), in which *Dgat1*^-/- ^mice require a ~20% higher glucose infusion rate than WT mice to maintain euglycemia [4]. Partial DGAT1 deficiency appears to increase insulin sensitivity as well, as suggested by decreased blood glucose levels after intraperitoneal injections of insulin in inbred DGAT1-heterozygous (*Dgat1*^+/-^) mice [6]. The increase in systemic insulin sensitivity likely can be attributed to increased insulin-stimulated glucose transport in the skeletal muscle and white adipose tissue (WAT) [7].

Although the precise molecular mechanisms that mediate these findings remain to be fully elucidated, insulin-stimulated activities of phosphatidylinositol-3 kinase, protein kinase B, and protein kinase Cλ, three key molecules in the insulin signaling pathway, are increased in the skeletal muscle of *Dgat1*^-/- ^mice. In addition, levels of serine-phosphorylated insulin receptor substrate-1, a molecule implicated in insulin resistance, are decreased in the skeletal muscle and WAT of *Dgat1*^-/- ^mice [7].

### Obesity resistance and increased energy expenditure in DGAT1-deficient mice

When fed a high-fat diet, *Dgat1*^-/- ^mice are resistant to weight gain and accumulation of adipose mass, and inbred *Dgat1*^+/- ^mice have an intermediate phenotype [1,6]. Because DGAT1 deficiency does not affect lean body mass, reductions in fat pad mass and tissue triglyceride levels most likely account for the differences in body weight [1].

The decreased adiposity and resistance to diet-induced obesity in *Dgat1*^-/- ^mice result from an increase in total energy expenditure [1]. The increased energy expenditure is attributable to at least two mechanisms - increased ambulatory physical activity in either chow (unpublished observations) or high-fat-fed conditions [1], and increased expression of uncoupling protein 1 [4], which is a major mediator of nonshivering thermogenesis in rodents.

Interestingly, *Dgat1*^-/- ^mice eat more than WT mice, and the hyperphagia is more pronounced during exposure to cold. When fasted in a cold environment, *Dgat1*^-/- ^mice develop hypothermia, which is associated with hypoglycemia. These results suggest that the hyperphagia in *Dgat1*^-/- ^mice is a secondary mechanism that compensates for the increased utilization of fuel substrates [8].

### Altered expression of adipocyte-secreted factors in DGAT1-deficient mice

How DGAT1 deficiency increases energy expenditure and insulin sensitivity remains incompletely understood. However, one contributing mechanism is the altered endocrine function of WAT in *Dgat1*^-/- ^mice. Recent studies have identified WAT as an important endocrine organ that regulates energy and glucose metabolism through secreted factors such as leptin, adiponectin, and resistin. Alterations in adipocyte size have been shown to correlate with changes in the endocrine function of WAT, and modulating DGAT1 expression affects adipocyte size in mice [4,9]. It is perhaps not surprising, then, that DGAT1 deficiency affects the expression and secretion of several adipocyte-derived factors that modulate energy and glucose metabolism [10].

**Figure 2:**
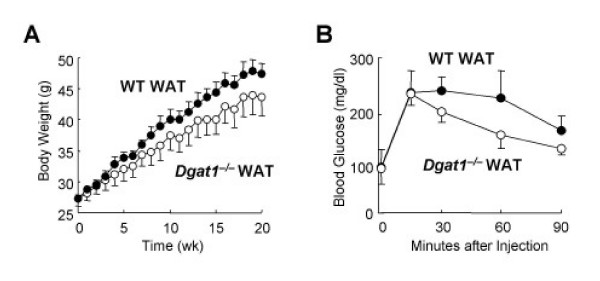
**Obesity resistance and enhanced glucose tolerance in WT mice transplanted with *Dgat1*^-/- ^WAT**. A. Body weight response to high-fat feeding. Mice were placed on a high-fat diet one week after fat transplantation. B. Glucose tolerance test. Reproduced with permission from [9].

To determine whether the altered expression of adipocyte-derived factors has physiological consequences, WAT from *Dgat1*^-/- ^mice was transplanted into WT recipient mice to assess its effects on glucose disposal and the response to a high-fat diet. Remarkably, transplantation of *Dgat1*^-/- ^WAT conferred partial obesity resistance (Figure 2A), enhanced glucose disposal after a glucose load (Figure 2B), and increased activation of the insulin signaling pathway in WT recipient mice [7,10]. In contrast, transplantation of WT adipose tissue into *Dgat1*^-/- ^mice did not adversely affect their resistance to diet-induced obesity or enhanced response to insulin. These results provide strong evidence that the altered endocrine function of *Dgat1*^-/- ^WAT has beneficial effects on systemic energy and glucose metabolism. Moreover, they suggest that these effects likely result from an increased secretion of adipocyte-derived factors by *Dgat1*^-/- ^WAT [10]. It will be of interest to better understand how DGAT1 deficiency alters the endocrine function of the WAT, and which factors contribute to the increased energy expenditure and insulin sensitivity in *Dgat1*^-/- ^mice.

### Perspectives and future directions

The *Dgat1*^-/- ^mouse is one of many genetically engineered murine models characterized by enhanced energy and glucose metabolism. For example, inactivation of other key components of the lipid synthesis pathway (*e.g*., acetyl CoA carboxylase 2, stearoyl CoA desaturase 1, and acylation stimulating protein) also results in obesity resistance and/or improved glucose metabolism in mice [11,12]. These results suggest the existence of a final common mechanism for increased energy expenditure, obesity resistance, and enhanced glucose disposal. It is unclear whether this common mechanism is mediated through the central nervous system (*e.g*., hypothalamus) or occurs directly in the peripheral tissues (*e.g*., adipose tissue, skeletal muscle). Given the complexity of regulating energy and glucose metabolism, however, it most likely involves multiple levels of interaction between the brain and the periphery.

Recent knockout mouse models have also provided insights into the mechanisms by which cellular lipid metabolism modulates systemic carbohydrate and insulin metabolism. The disruption of enzymes in the anabolic process of lipid uptake and storage (*e.g*., lipoprotein lipase, DGAT1) is more likely to enhance tissue glucose disposal or insulin secretion, whereas disruptions of enzymes in the catabolic process (*e.g*., hormone-sensitive lipase) tend to impair insulin action or secretion [13]. In these various knockout models, glucose disposal does not appear to have a strong correlation with levels of plasma free fatty acids, which have been hypothesized to be an important correlative of insulin resistance. Rather, these studies appear to support the notion that concentrations of fatty acid-derived molecules (*i.e*., fatty acyl CoAs, diacylglycerols) within cells, particularly myocytes, hepatocytes, and pancreatic β-cells, relate more directly to insulin resistance [14,15].

It is interesting that the disruption of lipid synthesis enzymes in mice achieves several outcomes that are typically associated with carbohydrate restriction [16]. Further research may help to determine whether mechanisms that mediate the metabolic effects of lipid synthesis inhibition also contribute to the beneficial effects of carbohydrate restriction. Additionally, further study of the mechanisms underlying the increased energy expenditure and increased insulin sensitivity in these mouse models may identify other novel therapeutic targets for human obesity and type 2 diabetes.

## Abbreviations

acyl CoA:diacylglycerol acyltransferase (DGAT), acyl CoA:monoacylglycerol acyltransferase (MGAT), white adipose tissue (WAT), wild type (WT)

## Competing interests

Although currently employed by Amgen Inc, the author conducted the DGAT1 studies reviewed in this article at the Gladstone Institutes and the University of California, San Francisco.
